# Exploring Different Sampling Strategies: A Description of Our Success in Reaching Hard‐to‐Reach Turkish and Moroccan Immigrant Women in The Netherlands

**DOI:** 10.1111/hex.70105

**Published:** 2024-12-15

**Authors:** Nora Hamdiui, Maartje Boer, Jim van Steenbergen, Maria van den Muijsenbergh, Aura Timen, Mart Stein

**Affiliations:** ^1^ Centre for Infectious Disease Control National Institute for Public Health and the Environment Bilthoven The Netherlands; ^2^ Department of Primary and Community Care Radboud University Medical Center Nijmegen The Netherlands; ^3^ Centre for Infectious Diseases Leiden University Medical Centre Leiden The Netherlands; ^4^ Pharos: Dutch Centre of Expertise on Health Disparities Program Prevention and Care Utrecht The Netherlands

**Keywords:** immigrants, Netherlands, peer‐to‐peer, questionnaire, reach, sampling

## Abstract

**Introduction:**

In the Netherlands, since 1996, there is a national cervical cancer (CC) screening programme in place for women aged 30–60 years. The participation of Turkish‐ and Moroccan‐Dutch women is very low. To facilitate their informed decision‐making, we developed a culturally sensitive educational video, and evaluated it through a questionnaire study. Since we used multiple strategies for the recruitment of respondents, we aimed (1) to explore which sampling strategy resulted in which type of respondents, (2) to investigate which sampling strategy and individual characteristics were associated with successful recruitment of other respondents, and (3) to examine similarity between those recruited via respondent‐driven sampling (RDS).

**Methods:**

Six sampling strategies were used and compared to explore their recruitment success: (1) RDS (i.e. peer‐to‐peer recruitment), (2) public and private women's Facebook groups, (3) Instagram, (4) researchers' network, (5) offline organizations (e.g., community centres and mosques), and (6) other channels (e.g. flyers, infographics, and information meetings). To do this, *χ* ^2^ tests, a multivariate logistic regression, and intra class correlations (ICCs) were performed.

**Results:**

Overall, 782 Moroccan‐ and 696 Turkish‐Dutch respondents were included in the analysis. Almost 40% filled out the questionnaire via RDS. RDS yields more often older, lower educated, and first‐generation immigrant women than average. Respondents recruited via RDS have more often low CC screening knowledge and make more often uninformed CC screening decisions than average. Social media channels, however, yielded more younger, highly educated, and second‐generation immigrant women than average. Sociodemographic characteristics and attitudes towards CC screening varied more strongly within than between network trees. The probability that paired respondents within a network tree had similar characteristics varied strongly depending on the characteristic.

**Conclusions:**

By using RDS and asking respondents to recruit peers, the more hard‐to‐reach individuals (i.e. older, lower educated, and first‐generation immigrants) were reached. By using social media channels, younger, highly educated, and second‐generation individuals can be recruited. RDS yielded more often women with low CC screening knowledge and women making uninformed CC screening decisions. To reach the individuals in need of tailored information or an intervention conform their needs, we recommend to use RDS as an intervention delivery strategy.

**Patient or Public Contribution:**

Following RDS, we involved Moroccan‐ and Turkish‐Dutch women in recruiting other Moroccan‐ and Turkish‐Dutch women. Through this recruitment, women were able to fill out our questionnaire, and watch our culturally sensitive educational video to improve their informed decision‐making in regard to the CC screening programme.

## 1 Introduction

In the Netherlands, since 1996, there has been a national cervical cancer (CC) screening programme in place for women aged 30–60 years. The overall participation rate was only 46% in 2022, while women with a Turkish or Moroccan migration background even showed lower participation rates [1, 2]. More worrying is that previous research has shown that these women do not make an informed decision regarding their participation, because of an overall lack of knowledge regarding CC (screening) and not being familiar with the possible personal disadvantages of CC screening (e.g., false‐positive or ‐negative test results, overdiagnosis and ‐treatment, and discomfort or pain) [3].

Turkish‐ and Moroccan‐Dutch women have shown to not only consider factual medical information, but also practical, emotional, cultural, and religious aspects before deciding whether to screen for CC [3]. However, the current information materials predominantly contain factual medical information. Therefore, we developed a culturally sensitive educational video (CSEV) with a specific focus on affective information, aiming to increase their informed decision‐making (IDM) regarding CC screening participation. The effect of the CSEV was evaluated on knowledge, attitude, intention, and IDM regarding CC screening among Turkish‐ and Moroccan‐Dutch women aged 30–60 years. These results can be found in our earlier publication [4].

Worldwide, our CSEV evaluation study (by means of a questionnaire) had one of the largest samples of Turkish‐ and Moroccan‐Dutch women successfully recruited. In this evaluation study, we used multiple strategies for the recruitment of respondents to fill out the questionnaire.

We used web‐based respondent‐driven sampling (RDS) as the main sampling strategy, as previous attempts have shown that traditional random sampling strategies are not effective in reaching these populations effectively [5] while their close‐knit social networks enable respondents to recruit each other easily [6].

RDS starts with a convenience sample of the population studied [7]. The first recruited individuals, the so‐called seeds (wave zero), are asked by the researchers to complete an online questionnaire and recruit other Turkish‐ and Moroccan‐Dutch women aged 30–60 years to fill out the same questionnaire. These recruited women represent Wave 1 and are asked the same, which may lead to Waves 2, 3, and so on. This process is repeated until the desired sample size or ‘equilibrium’ (i.e., when the sample characteristics are assumed to be independent from the seeds' characteristics) is reached, or if the peer recruitment dies out by itself. To follow who recruited whom, automatically generated unique codes are used and connections between individuals who recruited each other are drawn. A seed with all connected recruits forms one network tree.

In our CSEV evaluation study, other sampling strategies were used, besides RDS. These strategies included Facebook groups, Instagram influencers, the researchers' network (e.g., LinkedIn), offline organizations (e.g., community centres and mosques), and other channels (e.g., general practitioner practices, online infographics, and an online information meeting). Using these multiple sampling strategies, a diverse sample of Turkish‐ and Moroccan‐Dutch women was recruited. These women are usually underrepresented in scientific research and are considered hard‐to‐reach for public health institutes and researchers, as are other populations with non‐dominant mother tongue or cultural background, or with limited (health) literacy skills. Some subgroups (e.g., ≥ 40 year old, uneducated, and first‐generation immigrant women), however, were still not optimally reached [4].

However, during our CSEV evaluation study, we accomplished recruiting Turkish‐ and Moroccan‐Dutch women in relatively large numbers. The purpose of the present study was to fulfil the following three aims.

First, we explored which sampling strategy resulted in which type of respondents, in terms of sociodemographic characteristics, but also in terms of their knowledge, attitude, intention, and IDM regarding CC screening.

Second, we investigated which sampling strategy and individual characteristics were associated with successful recruitment of other respondents.

Third, we examined similarity between individuals stemming from the first recruited individual (i.e., seed) and similarity between recruiters and recruits in terms of sociodemographic characteristics and CC screening behaviour.

Our findings may help other researchers in their efforts to better recruit hard‐to‐reach populations for scientific research or more practical purposes (e.g. information or intervention delivery).

## 2 Methods

### 2.1 Study Design and Population

Between November 2020 and August 2021, we evaluated our developed CSEV with a control and an intervention group. The results of this evaluation can be found in our earlier publication [4]. In this evaluation study, respondents were included if the following criteria were met: (1) aged 30–60 years, (2) born in Turkey or Morocco and having at least one parent born in Turkey or Morocco (first‐generation immigrants), or born in the Netherlands and having at least one parent born in Turkey or Morocco (second‐generation immigrants), and (3) living in the Netherlands. This present study explores the sampling strategies that were used in the evaluation study, as described in the aims in the Introduction.

### 2.2 Sampling Strategies

We established six *sampling strategies* through which respondents were recruited: (1) RDS (i.e., recruited via their peer), (2) public and private women's groups on Facebook, (3) Instagram, (4) researchers' network (e.g., LinkedIn), (5) offline organizations (e.g., community centres and mosques), and (6) other channels (e.g., flyers at general practitioner practices, online infographics, and an online information meeting).

Following RDS (Strategy 1), respondents were asked to invite peers (i.e., women they know) to complete the same questionnaire. These invitations could be sent via WhatsApp, platforms such as Instagram, and/or SMS text messaging. In addition, reminders were sent by the researchers (whenever an email address was provided, which was optional) to complete and/or forward the questionnaire and to encourage respondents to remind their peers to complete the questionnaire (after 1 week of no participation of at least one peer). Initially, an incentive of €10 was given to every respondent who completed the questionnaire herself and peer‐recruited two other women who also completed the questionnaire. From March 2021, to further stimulate peer recruitment, an incentive of €15 was given to every respondent who completed the questionnaire herself and peer‐recruited one other woman who also completed the questionnaire.

Recruitment via public and private women's groups on Facebook (Strategy 2) was established by launching a post in these groups with information about the study (including a link to the questionnaire) and asking Turkish‐ and Moroccan‐Dutch women to fill out the questionnaire. To enable recruitment via Instagram (Strategy 3), we contacted several influencers with many Turkish‐ and Moroccan‐Dutch female followers and asked them to share the link to the questionnaire via their story or bio. A post was also launched on the researchers' LinkedIn pages (Strategy 4). This post included information about the study, a friendly request to participate in the study, and a link to the questionnaire to do so. Strategies 5 and 6 included a number of efforts, such as posters at community centres and mosques, flyers at general practitioner practices, online infographics shared by women who already participated in the study, and an information meeting held online by the researchers of this present study.

### 2.3 Questionnaire

We used a web‐based questionnaire that contained 52 questions regarding sociodemographic characteristics, previous CC screening participation, knowledge of CC screening, attitude towards CC screening, and intention to participate in the next CC screening round. It took women approximately 15 min to complete the questionnaire. For more information about the development and content of the questionnaire, our earlier publication can be consulted [4].

### 2.4 Statistical Analysis

#### 2.4.1 Study Measures

Several sociodemographic characteristics were assessed.

Respondent's *age* was calculated based on their birthdate and day of questionnaire participation. Respondents reported their highest obtained *educational level*, ranging from no education to higher vocational or university.

Respondents were classified as being either first‐ or second‐*generation* immigrant, based on their own and parents' country of birth.

Additionally, respondent's self‐reported CC screening behaviour was assessed, including their past *screening participation* (‘no, never’, ‘yes: every 5 years’, or ‘yes, but not every 5 years’) and screening participation through *self‐sampling* instead of clinician‐based sampling (‘yes’ or ‘no’).

Three items examined respondents' *knowledge* of CC screening, for example: ‘What should you do if the test result is good?’, of which the correct answer was: ‘Nothing, I will automatically receive a new screening invitation in five years’. Correct answers on all three questions yielded 4 points. Three or four points were classified as sufficient knowledge and 0–2 points as low knowledge. *Attitude towards screening* was measured using 10 items, for example: ‘Do you find it useful to do the screening?’, with response options (0) ‘no’ (1), ‘don't know’, and (2) ‘yes’. Scores were summed and multiplied by 5 to create scale scores ranging from 0 to 100. We distinguished negative (scores < 45), neutral (scores 45–55), and positive (scores > 55) attitudes. Also, respondent's *intention* to participate in the next screening opportunity was assessed, with response options ‘yes’, ‘no’, and ‘don't know’. Respondents' knowledge, attitude, and intention scores were used to calculate *IDM*. Those with sufficient knowledge and whose attitude towards screening matched their intention (i.e., both positive or negative), were regarded informed, and the remainder uninformed.

#### 2.4.2 Data Selection

We chose to perform our analyses for Moroccan‐ and Turkish‐Dutch women separately based on our experiences that each immigrant population has its own used platforms and specific needs. Therefore, to provide practical implications for each immigrant population specifically, analyses are done separately. Respondents who had terminated the questionnaire early were considered as refusing participation and therefore removed from the sample. Respondents not meeting the study's inclusion criteria with respect to age, migration background, and residential country were also removed. Also, responses from questionnaires were regarded as unreliable and therefore excluded whenever one of the following criteria was met: (1) the respondent and her recruit completed the questionnaire in < 5 min, or (2) the respondent or her recruit completed the questionnaire in < 5 min, and there was < 5 min between the start of the two participations. These criteria were defined, as it was practically impossible to read and answer the questions in less than 5 min. Most of the unreliable questionnaires were part of one network tree, which consists of the first recruited individual (i.e., seed) and all the individuals stemming from this seed. All members of this tree were additionally excluded from the analyses.

#### 2.4.3 Analytical Approach

First, to investigate whether different sampling strategies yielded different types of respondents (Aim 1), we studied bivariate associations between the sampling strategies and all study measures (see under Section 2.4.1) using *χ* ^2^ tests. Standardized residuals were consulted to study the source of significant *χ* ^2^ test, where absolute values of two or more were considered substantial.

Second, to study factors associated with the recruitment of other respondents (Aim 2), we studied bivariate associations between all study measures and successful recruitment using *χ* ^2^ tests. Recruitment was successful when it yielded at least one valid respondent completing the questionnaire. Next, for seeds and recruits separately, we conducted multivariate logistic regression to investigate whether socio‐demographic characteristics, screening participation, and informed CC screening decision‐making were associated with successful recruitment. For seeds, we also added the sampling strategy to the multivariate model. For recruits, a multivariate multilevel logistic model was adopted (Level 1 = respondents, Level 2 = network trees), with the associations modelled at the first level. Estimates from the logistic models were transformed into average predicted probabilities to facilitate the interpretation of the findings. Some of the covariates from the multivariate models were strongly associated. However, variance inflation factors of all covariates were less than two in the multivariate models, suggesting minimal multicollinearity [8].

**Figure 1:**
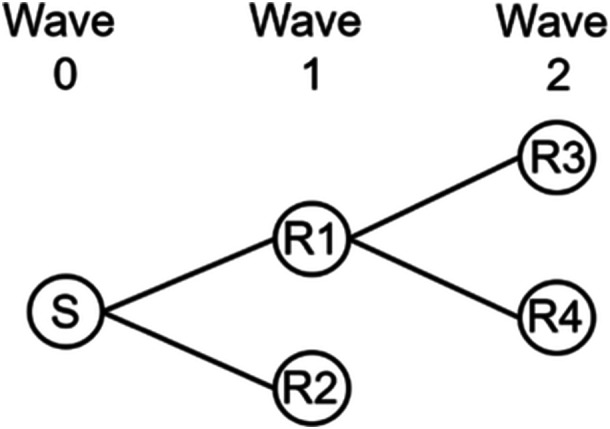
Illustration of a network tree. Diagram shows a network tree of five network members (circles) and two waves, including one seed (S) and four recruits (R1–R4). The network tree consists of four direct ties with tie distance one (S–R1; S–R2; R1–R3; R1–R4), four indirect ties with tie distance two (S–R3; S–R4; R1–R2; R3–R4), and two indirect ties with tie distance three (R2–R4; R2–R3).

Third, to explore similarity of respondents (Aim 3), we compared the variance of the study measures between network trees to the total variance of the study measures by computing intra class correlations (ICCs). The higher the ICC value (ranging from 0 to 1), the more respondents within a network tree were similar to each other. Next, data were restructured such that rows indicated pairs of respondents in the same network tree instead of individuals. For each pair, we computed their *tie distance* [9], ranging from ‘1’ (i.e., direct tie; recruiter and recruit) to ‘4 or more’ (i.e., indirect tie). Figure 1 explains how tie distance was calculated. To calculate this accurately, tie distances were computed using the sample with respondents meeting and not meeting all inclusion criteria. Also, we constructed variables indicating whether pairs of respondents had similar characteristics (e.g., *age similarity*: 1 = same age group, 0 = not the same age group). We then estimated the association between pairs' tie distance and similarity using bivariate multilevel logistic regression (Level 1 = pairs, Level 2 = network trees), with the association modelled at the first level. Also here, model estimates were transformed into average predicted probabilities. These associations reveal whether the similarity of respondents within a network tree depended on how closely they were linked to each other.

All *χ* ^2^ tests considered clustering by network trees, which means that the tests were adjusted for dependent observations within network trees. Logistic regression models were estimated using maximum likelihood. Also, logistic regression models were evaluated using likelihood ratio tests comparing the models with intercept‐only models, as well as McFadden's pseudo seudo, where values between 0.20 and 0.40 indicate good model fit [10]. Analyses were conducted using R version 4.2.2.

## 3 Results

The raw datafiles included 1607 Moroccan‐ and 1260 Turkish‐Dutch respondents. After data cleaning, these sample sizes reduced to 836 and 712, respectively. This reduction was mainly due to excluding respondents who had refused participation by terminating the questionnaire early (around 70%).

Within the analysis samples (*n*_Moroccan‐Dutch_ = 836, *n*_Turkish‐Dutch_ = 712), there were respondents with missing data on the sampling strategy due to technical deficits (*n*_Moroccan‐Dutch_ = 54, *n*_Turkish‐Dutch_ = 16). Their status as recruiter or recruit was unknown. Among respondents that participated in CC screening, data on self‐sampling were incomplete (*n*_Moroccan‐Dutch_ = 50, *n*_Turkish‐Dutch_ = 125). Data on other measures were complete. Cases with missing data were excluded from the analyses. This resulted in 782 Moroccan‐ and 696 Turkish‐Dutch respondents included in the analysis.

### 3.1 Sample Composition By Sampling Strategy (Aim 1)

In both samples (Moroccan‐ and Turkish‐Dutch), almost 40% filled out the questionnaire via their peer (i.e., through RDS) (see Table 1). Also, many Moroccan‐Dutch women participated via Facebook groups (35.55%), whereas Turkish‐Dutch women often participated via Instagram influencers (39.22%).

**Table 1:** Frequency and percentage of respondents per sampling strategy.

	Moroccan‐Dutch (*n*_individuals_ = 782)		Turkish‐Dutch (*n*_individuals_ = 696)	
**Recruited via**	**Frequency**	**Percentage (%)**	**Frequency**	**Percentage (%)**
Their peer (RDS)	305	39.00	266	38.22
Facebook group	278	35.55	67	9.63
Instagram influencers	77	9.85	273	39.22
Offline organizations	12	1.53	15	2.16
Researchers' network (LinkedIn)	79	10.10	66	9.48
Other	31	3.96	9	1.29

Abbreviation: RDS, respondent‐driven sampling (i.e., recruited via their peer).

Moroccan‐ and Turkish‐Dutch respondents that were recruited via RDS were more often older, lower educated, and first‐generation immigrant women than average (see Tables 2a and 2b). As an example, 15.41% of the Moroccan‐Dutch respondents was 50 years or older in the RDS sample, while this percentage was much lower for the other samples (5.40% Facebook, 1.30% Instagram, 3.80% LinkedIn, and 13.95% Offline/Other). Social media channels Facebook and Instagram attracted more younger and highly educated respondents. The Moroccan‐Dutch sample recruited via Facebook consisted of 62.23% respondents with a higher vocational/university education, in comparison to a percentage of 38.49% in the RDS sample. Social media also reached more second‐generation immigrant women, although within the Turkish‐Dutch sample respondents participating via Facebook were relatively often of the first generation. Moroccan‐Dutch respondents sampled via the researchers' network were often highly educated.

**Table 2a:** Demographic characteristics, by sampling strategy (Moroccan‐Dutch sample, *n*_individuals_ = 782).

	Total		RDS		Facebook group		Instagram influencers		Researchers' network (LinkedIn)		Offline/other	
	***N***	**%**	***n***	**%**	***n***	**%**	***n***	**%**	***n***	**%**	***n***	**%**
Age***												
30–39	435	55.63	*143*	*46.89*	**170**	**61.15**	**56**	**72.73**	45	56.96	21	48.84
40–49	275	35.17	115	37.70	93	33.45	20	25.97	31	39.24	16	37.21
50+	72	9.21	**47**	**15.41**	*15*	*5.40*	*1*	*1.30*	3	3.80	6	13.95
Educational level***												
No education, primary school, or high school	176	22.54	**101**	**33.22**	*40*	*14.39*	12	15.58	12	15.19	11	25.58
Vocational school	182	23.30	**86**	**28.29**	65	23.38	15	19.48	*7*	*8.86*	9	20.93
Higher vocational/university	423	54.16	*117*	*38.49*	**173**	**62.23**	50	64.94	**60**	**75.95**	23	53.49
Generation***												
First	319	40.79	**157**	**51.48**	*98*	*35.25*	*18*	*23.38*	25	31.65	21	48.84
Second	463	59.21	*148*	*48.52*	**180**	**64.75**	**59**	**76.62**	54	68.35	22	51.16
Screening participation												
No, never	290	37.08	128	41.97	94	33.81	22	28.57	30	37.97	16	37.21
Yes, every 5 years	383	48.98	138	45.25	139	50.00	44	57.14	41	51.90	21	48.84
Yes, but not every 5 years	109	13.94	39	12.79	45	16.19	11	14.29	8	10.13	6	13.95
Self‐sampling												
No	347	78.33	124	78.98	128	75.29	35	72.92	41	91.11	19	82.61
Yes	96	21.67	33	21.02	42	24.71	13	27.08	4	8.89	4	17.39
CC screening knowledge***												
Low	268	34.27	**136**	**44.59**	*75*	*26.98*	27	35.06	*14*	*17.72*	16	37.21
Sufficient	514	65.73	*169*	*55.41*	**203**	**73.02**	50	64.94	**65**	**82.28**	27	62.79
Attitude												
Negative	87	11.13	43	14.10	21	7.55	7	9.09	9	11.39	7	16.28
Neutral	171	21.87	66	21.64	61	21.94	18	23.38	16	20.25	10	23.26
Positive	524	67.01	196	64.26	196	70.50	52	67.53	54	68.35	26	60.47
Intention												
No	41	5.24	15	4.92	15	5.40	3	3.90	7	8.86	1	2.33
Don't know	113	14.45	59	19.34	32	11.51	7	9.09	8	10.13	7	16.28
Yes	628	80.31	231	75.74	231	83.09	67	87.01	64	81.01	35	81.40
Informed decision‐making***												
Informed	361	46.16	*113*	*37.05*	**151**	**54.32**	35	45.45	**45**	**56.96**	17	39.53
Uninformed	421	53.84	**192**	**62.95**	*127*	*45.68*	42	54.55	*34*	*43.04*	26	60.47

*Note: p* values from *χ* ^2^ test. Standardized residuals were inspected to find the source of significant *χ* ^2^ tests. Standardized residuals of two or higher (in boldface) or minus two or lower (in italics) indicate that the observed frequencies were higher or lower than expected.

Abbreviation: RDS, respondent‐driven sampling (i.e., recruited via their peer).

*** *p* < 0.001.

**Table 2b:** Demographic characteristics, by sampling strategy (Turkish‐Dutch sample, *n*_individuals_ = 696).

	Total		RDS		Facebook group		Instagram influencers		Researchers' network (LinkedIn)		Offline/other	
	***N***	**%**	***n***	**%**	***n***	**%**	***n***	**%**	***n***	**%**	***n***	**%**
Age***												
30–39	421	60.49	*135*	*50.75*	42	62.69	**195**	**71.43**	36	54.55	13	54.17
40–49	184	26.44	76	28.57	19	28.36	61	22.34	19	28.79	9	37.50
50+	91	13.07	**55**	**20.68**	6	8.96	*17*	*6.23*	11	16.67	2	8.33
Educational level***												
No education, primary school, or high school	199	28.59	**101**	**37.97**	14	20.90	*60*	*21.98*	20	30.30	4	16.67
Vocational school	202	29.02	72	27.07	*11*	*16.42*	**95**	**34.80**	18	27.27	6	25.00
Higher vocational/university	295	42.39	*93*	*34.96*	**42**	**62.69**	118	43.22	28	42.42	14	58.33
Generation***												
First	287	41.24	**126**	**47.37**	**46**	**68.66**	*75*	*27.47*	29	43.94	11	45.83
Second	409	58.76	*140*	*52.63*	*21*	*31.34*	**198**	**72.53**	37	56.06	13	54.17
Screening participation**												
No, never	270	38.79	95	35.71	26	38.81	114	41.76	32	48.48	*3*	*12.50*
Yes, every 5 years	315	45.26	122	45.86	27	40.30	129	47.25	23	34.85	14	58.33
Yes, but not every 5 years	111	15.95	49	18.42	14	20.90	*30*	*10.99*	11	16.67	7	29.17
Self‐sampling												
No	244	81.06	105	78.95	14	87.50	90	80.36	26	89.66	9	81.82
Yes	57	18.94	28	21.05	2	12.50	22	19.64	3	10.34	2	18.18
CC screening knowledge												
Low	337	48.42	128	48.12	28	41.79	133	48.72	32	48.48	16	66.67
Sufficient	359	51.58	138	51.88	39	58.21	140	51.28	34	51.52	8	33.33
Attitude												
Negative	54	7.76	20	7.52	3	4.48	26	9.52	3	4.55	2	8.33
Neutral	158	22.70	59	22.18	14	20.90	54	19.78	25	37.88	6	25.00
Positive	484	69.54	187	70.30	50	74.63	193	70.70	38	57.58	16	66.67
Intention												
No	30	4.31	13	4.89	4	5.97	6	2.20	4	6.06	3	12.50
Don't know	120	17.24	46	17.29	9	13.43	43	15.75	17	25.76	5	20.83
Yes	546	78.45	207	77.82	54	80.60	224	82.05	45	68.18	16	66.67
Informed decision‐making												
Informed	255	36.64	96	36.09	29	43.28	105	38.46	20	30.30	5	20.83
Uninformed	441	63.36	170	63.91	38	56.72	168	61.54	46	69.70	19	79.17

*Note: p* values from *χ* ^2^ test. Standardized residuals were inspected to find the source of significant *χ* ^2^ tests. Standardized residuals of two or higher (in boldface) or minus two or lower (in italics) indicate that the observed frequencies were higher or lower than expected.

Abbreviation: RDS, respondent‐driven sampling (i.e., recruited via their peer).

** *p* < 0.01

*** *p* < 0.001.

With regard to CC screening behaviour, asking respondents to invite other respondents yielded relatively many Moroccan‐Dutch women with low CC screening knowledge (44.59%) and uninformed CC screening decision‐making (62.95%). By contrast, Moroccan‐Dutch women participating via Facebook and the researchers' network more often reported sufficient CC screening knowledge (73.02% and 82.28%, respectively) and informed CC screening decision‐making (54.32% and 56.96%, respectively). Within the Turkish‐Dutch sample, there were relatively few Instagram‐respondents that irregularly participated in the CC screening (i.e., not every 5 years), and relatively few offline/other‐respondents that never participated in the CC screening. No other statistically significant associations were found between CC screening behaviour and the sampling strategy, although this could be related to limited power, because relatively few respondents participated via offline/other‐channels.

### 3.2 Associations With Successful Recruitment (Aim 2)

In both samples (Moroccan‐ and Turkish‐Dutch), women participating via the researchers' network were less likely to recruit than women participating via other strategies (see Table 3). Within the Moroccan‐Dutch sample, recruiting occurred most often among women participating via offline organizations or other routes (44.19% recruited). Also, Moroccan‐Dutch women that did not know whether they would participate in the next CC screening round recruited relatively often (39.17% recruited). Moroccan‐Dutch women that irregularly participated in CC screening (16.52% recruited) or who intended to participate in the next CC screening round (23.15% recruited) were less likely to recruit. Within the Turkish‐Dutch sample, successful recruitment was associated with other factors: women from the second generation (26.56% recruited) or with sufficient knowledge about CC screening (27.12% recruited) were most likely to recruit.

**Table 3:** Successful recruitment, by demographic characteristics and (attitude toward) CC screening.

	Moroccan‐Dutch sample				Turkish‐Dutch sample			
	*n* Total	*n* Recruiters	% Recruiters	*p* value	*n* Total	*n* Recruiters	% Recruiters	*p* value
Sampling strategy								
Recruited via their peer (RDS)	305	90	29.51	0.187	266	70	26.32	0.169
Facebook groups	278	61	21.94	0.206	67	20	29.85	0.166
Instagram influencers	77	16	20.78	0.378	273	58	21.25	0.350
Researchers' network (LinkedIn)	79	11	13.92	0.027*	66	6	9.09	0.006**
Offline organizations/other	43	19	44.19	0.005**	24	7	29.17	0.483
Age								
30–39	459	114	24.84	0.699	428	97	22.66	0.785
40–49	298	75	25.17	0.882	190	48	25.26	0.434
50+	79	24	30.38	0.451	94	19	20.21	0.573
Educational level								
No education, primary school, or high school	189	51	26.98	0.694	204	41	20.10	0.199
Vocational school	202	55	27.23	0.535	209	51	24.40	0.533
Higher vocational/university	444	107	24.10	0.457	299	72	24.08	0.555
Generation								
First	349	86	24.64	0.713	294	53	18.03	0.013*
Second	487	127	26.08	0.713	418	111	26.56	0.013*
Screening participation								
No, never	305	88	28.85	0.131	274	61	22.26	0.680
Yes, every 5 years	416	106	25.48	0.999	324	79	24.38	0.441
Yes, but not every 5 years	115	19	16.52	0.030*	114	24	21.05	0.576
Self‐sampling								
No	373	88	23.59	0.469	253	64	25.30	0.959
Yes	108	22	20.37	0.469	60	15	25.00	0.959
CC screening knowledge (pre)								
Low	287	78	27.18	0.533	347	65	18.73	0.014*
Sufficient	549	135	24.59	0.533	365	99	27.12	0.014*
Attitude (pre)								
Negative	96	21	21.88	0.409	55	14	25.45	0.668
Neutral	187	51	27.27	0.507	163	45	27.61	0.125
Positive	553	141	25.50%	0.986	494	105	21.26	0.092
Intention (pre)								
No	42	10	23.81	0.825	31	8	25.81	0.745
Don't know	120	47	39.17	0.002**	122	31	25.41	0.480
Yes	674	156	23.15	0.013*	559	125	22.36	0.406
Informed decision‐making (pre)								
Informed	383	89	23.24	0.234	259	65	25.10	0.343
Uninformed	453	124	27.37	0.234	453	99	21.85	0.343

*Note:* Recruiters were those that have recruited at least one valid respondent (i.e., not meeting any exclusion criteria) successfully. *p* values were calculated based on *χ* ^2^ tests comparing the proportion of recruiters within a particular group (i.e., participation through Facebook groups) versus the proportion of recruiters within all other groups.

Abbreviation: RDS, respondent‐driven sampling (i.e., recruited via their peer).

* *p* < 0.05

** *p* < 0.01.

**Figure 2:**
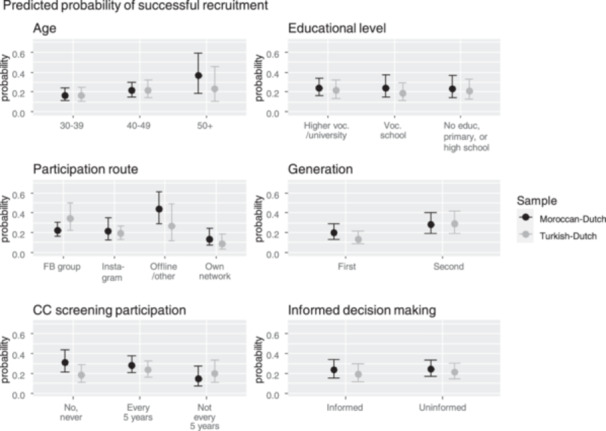
Average predicted probability of recruiting at least one valid respondent. Based on model estimates from Table 4.

Table 4 shows the results from the multivariate models estimated among seeds. Although according to the likelihood‐ratio tests the models fit better than models without any predictors, pseudo *R* ^2^ values were low, suggesting low explained variance. Within the Moroccan‐Dutch sample, seeds aged 50 years and older (OR of 2.865) and seeds participating via offline or other routes (OR of 2.739) showed a higher probability to recruit than seeds aged 30–39 and seeds participating via Facebook, respectively. Moroccan‐Dutch seeds participating in CC screening irregularly were less likely to recruit than Moroccan‐Dutch seeds that have never participated in CC screening (OR of 0.381). However, according to the 95% confidence intervals of the average predicted recruitment probabilities (see Figure 2), these found group differences were not statistically significant. Within the Turkish‐Dutch sample, second‐generation seeds showed a higher probability to recruit than first‐generation seeds (OR of 2.601), although the average predicted recruitment probabilities by generation did not significantly differ statistically. Model fit of the multivariate models among recruits was poor, and none of the study measures were statistically significantly associated with recruitment (see Supporting Information S1: Table A1).

**Table 4:** Multivariate logistic regression, recruiting at least one valid respondent.

	Moroccan‐Dutch sample, seeds (*n*_individuals_ = 477)				Turkish‐Dutch sample, seeds (*n*_individuals_ = 430)			
	Estimate	SE	*p*	OR	Estimate	SE	*p*	OR
Intercept	−1.560***	0.389	< 0.001		−1.516***	0.429	< 0.001	
Age (reference = 30–39)								
40–49	0.309	0.273	0.257	1.363	0.341	0.306	0.265	1.407
50+	1.053*	0.525	0.045	2.865	0.449	0.573	0.433	1.567
Educational level (reference = higher vocational/university)								
Vocational school	0.010	0.289	0.974	1.010	−0.180	0.292	0.536	0.835
No education, primary school, or high school	−0.031	0.340	0.927	0.970	−0.027	0.363	0.941	0.974
Generation (reference = first generation)								
Second generation	0.478	0.289	0.098	1.614	0.956**	0.325	0.003	2.601
Sampling strategy (reference = Facebook groups)								
Instagram influencers	−0.056	0.324	0.863	0.945	−0.805	0.341	0.018	0.447
Offline/other	1.007**	0.350	0.004	2.739	−0.366	0.556	0.511	0.694
Own network	−0.620	0.361	0.086	0.538	−1.715	0.526	0.001	0.180
Screening participation (reference = no, never)								
Yes, every 5 years	−0.149	0.276	0.588	0.861	0.310	0.292	0.287	1.364
Yes, but not every 5 years	−0.964*	0.423	0.023	0.381	0.111	0.405	0.783	1.118
Informed decision‐making (reference = informed)								
Uninformed	0.019	0.255	0.942	1.019	0.125	0.273	0.648	1.133
LR‐test *χ* ^2^	24.320*		0.011		22.174*		0.023	
Pseudo *R* ^2^	0.048				0.050			

Abbreviations: OR = odds ratio, *p* = *p* value, SE = standard error.

* *p* < 0.05

** *p* < 0.01

*** *p* < 0.001.

### 3.3 Similarity of Respondents (Aim 3)

We initially identified 190 Moroccan‐ and 143 Turkish‐Dutch network trees. The number of valid trees, that is, with at least two respondents meeting all inclusion criteria, was 128 and 98, respectively. From the Moroccan‐Dutch valid trees, 66 consisted of one wave and 47 of two waves, and the maximum number of waves was 18. From the valid Turkish‐Dutch trees, 60 consisted of one wave and 19 of two waves, and the maximum number of waves was eight.

The values of the study measures varied more strongly within than between network trees, as all ICCs were below 0.50 (see Table 5). However, in the Moroccan‐Dutch sample, all measures except for recruitment showed an ICC of 0.10 or higher. This indicates substantial variation across network trees and some degree of similarity on the study measures within network trees. In the Turkish‐Dutch network trees, study respondents in the same tree were less similar to each other, as half of the study measures had rather low ICCs (screening participation, self‐sampling, CC screening knowledge, informed CC screening decision‐making, and recruitment).

**Table 5:** Intra class correlations of the study measures.

	Moroccan‐Dutch (*n*_individuals_ = 423 within 128 network trees)^a^	Turkish‐Dutch (*n*_individuals_ = 352 within 98 network trees)^a^
Age	0.244	0.131
Educational level	0.179	0.135
Generation	0.338	0.272
Screening participation^b^	0.286	< 0.001
Self‐sampling^c^	0.162	< 0.001
CC screening knowledge	0.109	0.050
Attitude	0.208	0.170
Intention^d^	0.286	0.198
Informed decision‐making	0.168	0.093
Successful recruitment	0.053	0.027

*Note:* Intra class correlations were computed using between‐tree variance/(between‐tree variance + (*π* ^2^/3)). Between‐tree variances were derived from two‐level logistic (generation, screening participation, self‐sampling, CC screening knowledge, intention, informed decision making) and ordered logistic models (age, educational level, attitude).

^a^ Samples include those not meeting any exclusion criteria and who are member of a tree with at least two valid respondents.

^b^ Screening participation was recoded as (0) ‘no’, never versus (1) ‘yes (but not) every 5 years’.

^c^ Moroccan‐Dutch sample = 224 within 100 trees; Turkish‐Dutch sample = 173 within 69 trees.

^d^ Intention was recoded as (0) ‘no/don't know’ versus (1) ‘yes’.

**Figure 3:**
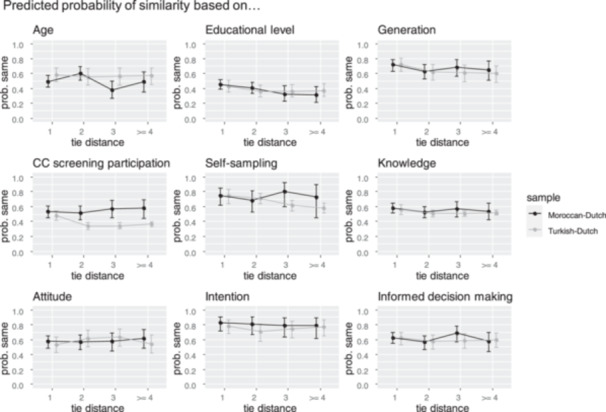
Estimated probability similarity. Based on model estimates from Supporting information S1: Table A2.

Restructuring the data from individuals to pairs and excluding pairs with respondents not meeting all inclusion criteria, the Moroccan‐Dutch sample counted 286 direct ties and 708, 130, and 200 indirect ties with tie distances two, three, and four or more, respectively. For the Turkish‐Dutch sample, these counts were 221, 435, 553, and 779, respectively. In both samples, the probability that paired respondents had similar characteristics generally remained stable with increasing tie distance (see Supporting information S1: Table A2). A few small differences by tie distance were observed (pseudo *R* ^2^ < 0.011). Among Moroccan‐Dutch ties, similarity based on educational level and generation decreased somewhat with increasing tie distance. Among Turkish‐Dutch ties, there were small decreasing trends for similarity based on generation, screening participation, and self‐sampling. Figure 3 shows that the probability of paired respondents to have similar characteristics varied from 31.09% (educational level) to 83.18% (intention).

## 4 Discussion

### 4.1 Main Findings

In this study, we explored which sampling strategy resulted in which type of respondents, in terms of sociodemographic characteristics, but also in terms of their knowledge, attitude, intention, and IDM regarding CC screening (Aim 1). We also investigated which sampling strategy and individual characteristics were associated with successful recruitment of other respondents (Aim 2). Finally, we examined similarity between tree members and similarity between recruiters and recruits in terms of sociodemographic characteristics and CC screening behaviour (Aim 3).

We found that RDS is the most successful sampling strategy in both Moroccan‐ and Turkish‐Dutch. We have also seen that RDS is feasible, as almost 40% of the samples (Moroccan‐ and Turkish‐Dutch) were recruited by their peer. The other 60% were recruited through Facebook groups, Instagram influencers, the researchers' network (e.g., LinkedIn), offline organizations (e.g., community centres and mosques), and other channels. The results also indicate that RDS yields more often older, lower educated, and first‐generation immigrant women than average. We have also seen that respondents recruited via RDS have more often low CC screening knowledge and make more often uninformed CC screening decisions than average. An explanation for this finding could be that RDS is a more personal method that could be attractive to individuals who are normally less likely to participate in research, but who are probably also less reached by information materials on CC screening. It is likely that RDS helped in reaching and including individuals who are not reached by the information materials of the Dutch National Institute of Public Health and the Environment, and therefore have a low CC screening knowledge and show uninformed CC screening decisions. Social media channels, however, yielded more younger, highly educated, and second‐generation immigrant women than average. RDS or any other similar peer recruitment strategy can thus be helpful in reaching populations researchers or policymakers are particularly interested in, such as first‐generation immigrant women with low knowledge about CC screening and women who make uninformed CC screening decisions.

Factors associated with successful recruitment differed for Moroccan‐ and Turkish‐Dutch women. Moroccan‐Dutch women participating via offline organizations or other routes, and those who did not know whether they would participate in the next CC screening round, recruited relatively often. Turkish‐Dutch women from the second generation or with sufficient knowledge about CC screening were most likely to recruit. In both Moroccan‐ and Turkish‐Dutch women, respondents participating via LinkedIn were less likely to recruit. This might be due to a smaller role of the financial incentive in this group. However, the multivariate models showed weak evidence for associations between the investigated characteristics and successful recruitment. Hence, more research exploring the determinants of recruitment is warranted.

In both samples (Moroccan‐ and Turkish‐Dutch), individuals within a network tree showed substantial differences, as their sociodemographic characteristics and attitudes towards CC screening varied more strongly within than between network trees. Also, the probability that paired respondents within a network tree had similar characteristics varied strongly, depending on the characteristic (e.g., educational level < 50%, intention to participate in CC screening > 80%). Together, these findings underline a certain degree of diversity within network trees. In both samples, the probability that paired respondents within a network tree had (dis)similar characteristics generally remained stable with increasing tie distance. In other words, direct and indirect ties were often equally likely to have (dis)similar characteristics. The finding that the probability of, for example, uninformed individuals successfully recruiting other uninformed individuals remains stable as recruiting continues, and thus does not decrease, underlines the relevance of using RDS and efforts to ‘grow’ network trees to reach individuals at interest.

### 4.2 Comparison With Previous Studies

A study in Mexico compared respondents (cisgender men who have sex with men [MSM] and transgender women) recruited via RDS versus venue‐based sampling [11]. Similar to our study in which RDS yielded the more hard‐to‐reach individuals (e.g., older, lower educated, more often uninformed), this Mexican study reported more injection drug use, higher levels of internalized stigma, and more often a history of deportation from the United States in the RDS sample.

Since in Canada francophones living outside the primarily French‐speaking province of Quebec risk being excluded from research, a previous study tried to recruit these individuals using RDS [12]. In line with the present study, compared to advertising, RDS yielded the more hard‐to‐reach individuals, represented by more males, immigrants, and those not having a regular medical doctor.

Another Canadian study aimed to recruit ‘mainstream’ marijuana users, as there is limited research about marijuana use among socially integrated individuals who hold jobs, raise families, and exhibit stable lifestyles [13]. In this study, similar to our study that reached the more hard‐to‐reach individuals through RDS, the RDS sample included twice as many female marijuana users as the sample that was derived by Random Digit Dialing (RDD). The RDS sample also yielded more individuals who completed university or college compared to the RDD sample.

In contrast with the present study, however, in a previous study in the United States, a higher HIV prevalence was found among MSM in the direct recruitment sample compared to the RDS sample [14]. Also, direct recruitment resulted in more Black respondents and those with less than a high school diploma, compared to RDS. Additionally, the prevalence of low income, no healthcare coverage, bisexuality, and hidden sexuality increased across RDS waves, while in our study, the study measures remained stable with increasing tie distance.

These differences may be explained by the specific ‘high‐risk’ sites, where the direct recruitment occurred, namely targeted community testing events (e.g., testing in parking lots of gay clubs), partnerships with HIV testing agencies, clinics, hospitals, support groups, community‐based organizations, and social media (e.g., by placing ads on Facebook and gay dating apps, such as Grindr).

### 4.3 Strengths and Limitations

Worldwide, this study had one of the largest samples successfully recruited using web‐based RDS [15]. Using different statistical methods, we also managed to gain detailed insights regarding the efficacy of RDS in reaching hard‐to‐reach Turkish‐ and Moroccan‐Dutch immigrant women in the Netherlands.

However, the number of individuals recruited through offline organizations, the researchers' network, and other channels was limited. In the future, larger samples recruited through these routes should be included to better estimate associations between these participation routes and study measures like CC screening behaviour and informed CC screening decision‐making.

### 4.4 Practical Implications

In line with previous literature, RDS is a successful sampling strategy to recruit especially the more hard‐to‐reach individuals. However, it is important to note the importance of using a guaranteed material incentive, since a previous scoping review found that studies that did not offer at least one guaranteed material incentive reported relatively fewer waves and lower percentages of successfully recruiting participants [15]. In the present study, enabling recruitment by peers yielded more often women with low knowledge about CC screening and women who make uninformed CC screening decisions. To reach the individuals in need of tailored information or an intervention conform their needs, we recommend to use RDS not only as a sampling strategy for filling out a questionnaire, but also as an intervention delivery strategy (e.g., by delivering our CSEV as previously done and described in our earlier publication [4].

## 5 Conclusions

To conclude, by using RDS and asking respondents to recruit peers, the more hard‐to‐reach individuals (i.e., older, lower educated, and first‐generation immigrants) were recruited and participated in the online questionnaire. By using social media channels, younger, highly educated, and second‐generation individuals can be recruited. Peer recruitment yielded more often women with low knowledge about CC screening and women making uninformed CC screening decisions. To reach the individuals in need of tailored information or an intervention conform their needs, we recommend to use RDS as an intervention delivery strategy.

## Author Contributions

**Nora Hamdiui:** writing–original draft, writing–review and editing, data curation, methodology, software, formal analysis, conceptualization, investigation, project administration, funding acquisition. **Maartje Boer:** formal analysis, writing–original draft, writing–review and editing. **Jim van Steenbergen:** conceptualization, supervision, writing–review and editing, methodology. **Maria van den Muijsenbergh:** conceptualization, methodology, writing–review and editing, supervision. **Aura Timen:** conceptualization, methodology, writing–review and editing, supervision, funding acquisition. **Mart Stein:** conceptualization, methodology, software, supervision, writing–review and editing, funding acquisition, project administration.

## Ethics Statement

The Medical Ethics Review Committee of the University Medical Centre Utrecht confirmed that the Medical Research Involving Human Subjects Act does not apply to this study (nr: 20/105). Respondents were informed about the subject and aims of the study, and were asked to give their informed consent digitally before filling in the questionnaire.

## Conflicts of Interest

The authors declare no conflicts of interest.

## Supporting information

Supporting information.

## Data Availability

The data that support the findings of this study are available on request from the corresponding author. The data are not publicly available due to privacy or ethical restrictions.
