# Supplementary material for: Exploring Different Sampling Strategies: A Description of Our Success in Reaching Hard‐to‐Reach Turkish and Moroccan Immigrant Women in The Netherlands
Source: Health Expect. 2024 Dec 15;27(6):e70105. doi: 10.1111/hex.70105 (PMC11647058; doi:10.1111/hex.70105)
Supplement: Supplementary file 1 — Supporting information. [file HEX-27-e70105-s001.docx]

**Exploring different sampling strategies: a description of our success in reaching hard-to-reach Turkish and Moroccan immigrant women in the Netherlands**

**Supporting information**

| ***Table A1.***  **Multivariate multilevel logistic regression, recruiting at least one valid respondent** | | | | | | | | |
| --- | --- | --- | --- | --- | --- | --- | --- | --- |
|  | **Moroccan-Dutch sample,**  **recruits (*n*_individuals_ = 305 within 132 trees)** | | | | **Turkish-Dutch sample,**  **recruits (*n*_individuals_ = 266 within 107 trees)** | | | |
|  | **Estimate** | ***SE*** | ***p*** | ***OR*** | **Estimate** | ***SE*** | ***p*** | ***OR*** |
| (Intercept) | -1.528** | 0.551 | 0.006 |  | -1.075* | 0.480 | 0.025 |  |
| Age (ref. = 30-39) |  |  |  |  |  |  |  |  |
| Age 40-49 | 0.347 | 0.373 | 0.352 | 1.415 | 0.361 | 0.366 | 0.324 | 1.435 |
| Age 50+ | -0.114 | 0.565 | 0.840 | 0.892 | 0.592 | 0.534 | 0.268 | 1.807 |
| Educational level (ref. = higher vocational / university) |  |  |  |  |  |  |  |  |
| Vocational school | 0.308 | 0.380 | 0.418 | 1.360 | 0.268 | 0.370 | 0.468 | 1.308 |
| No education, primary school, or high school | -0.186 | 0.411 | 0.651 | 0.830 | -0.172 | 0.403 | 0.669 | 0.842 |
| Generation (ref. = first generation) |  |  |  |  |  |  |  |  |
| Second generation | 0.306 | 0.370 | 0.408 | 1.358 | 0.475 | 0.381 | 0.213 | 1.608 |
| Screening participation (ref. = no, never) |  |  |  |  |  |  |  |  |
| Yes, every 5 years | -0.191 | 0.368 | 0.604 | 0.826 | -0.355 | 0.363 | 0.329 | 0.701 |
| Yes, but not every five years | -0.156 | 0.516 | 0.763 | 0.856 | -0.383 | 0.440 | 0.385 | 0.682 |
| Informed decision-making (ref. = informed) |  |  |  |  |  |  |  |  |
| Uninformed | 0.118 | 0.350 | 0.735 | 1.126 | -0.413 | 0.324 | 0.203 | 0.662 |
| SD random intercept | 0.981*** |  | < 0.001 |  | 0.388 |  | 0.290 |  |
| LR-test chi-square | 4.533 |  | 0.806 |  | 6.526 |  | 0.589 |  |
| Pseudo R-square | 0.013 |  |  |  | 0.021 |  |  |  |
| * *p* < 0.05, ** *p* < 0.01, *** *p* < 0.001. *SE* = standard error, *p* = *p*-value, *OR* = odds ratio. | | | | | | | | |

| ***Table A2.***  **Univariate multilevel logistic regression, similarity** | | | | | | | | |
| --- | --- | --- | --- | --- | --- | --- | --- | --- |
|  | **Moroccan-Dutch sample**  **(*n*_pairs_ = 1324 within 128 trees)** | | | | **Turkish-Dutch sample**  **(*n*_pairs_ = 1988 within 98 trees)** | | | |
| **Same age** | **Estimate** | ***SE*** | ***p*** | ***OR*** | **Estimate** | ***SE*** | ***p*** | ***OR*** |
| Tie distance 2 | 0.443* | 0.192 | 0.021 | 1.557 | -0.118 | 0.210 | 0.575 | 0.889 |
| Tie distance 3 | -0.473 | 0.256 | 0.065 | 0.623 | -0.105 | 0.216 | 0.627 | 0.900 |
| Tie distance 4 | -0.027 | 0.269 | 0.919 | 0.973 | -0.066 | 0.215 | 0.760 | 0.937 |
| SD random intercept | 0.955*** |  | < 0.001 |  | 1.116*** |  | < 0.001 |  |
| LR-test chi-square | 19.240*** |  | < 0.001 |  | 0.450 |  | 0.930 |  |
| Pseudo R-squared | 0.011 |  |  |  | < 0.001 |  |  |  |
| **Same educational level** | **Estimate** | ***SE*** | ***p*** | ***OR*** | **Estimate** | ***SE*** | ***p*** | ***OR*** |
| Tie distance 2 | -0.204 | 0.178 | 0.251 | 0.815 | -0.278 | 0.199 | 0.161 | 0.757 |
| Tie distance 3 | -0.541* | 0.239 | 0.024 | 0.582 | -0.269 | 0.205 | 0.189 | 0.764 |
| Tie distance 4 | -0.609* | 0.255 | 0.017 | 0.544 | -0.227 | 0.204 | 0.265 | 0.797 |
| SD random intercept | 0.513*** |  | < 0.001 |  | 0.534 |  | 0.549 |  |
| LR-test chi-square | 8.481* |  | 0.037 |  | 2.124 |  | 0.547 |  |
| Pseudo R-squared | 0.005 |  |  |  | 0.001 |  |  |  |
| **Same generation** | **Estimate** | ***SE*** | ***p*** | ***OR*** | **Estimate** | ***SE*** | ***p*** | ***OR*** |
| Tie distance 2 | -0.406* | 0.191 | 0.033 | 0.666 | -0.475* | 0.214 | 0.026 | 0.622 |
| Tie distance 3 | -0.147 | 0.244 | 0.547 | 0.863 | -0.527* | 0.219 | 0.016 | 0.591 |
| Tie distance 4 | -0.327 | 0.270 | 0.226 | 0.721 | -0.578** | 0.219 | 0.008 | 0.561 |
| SD random intercept | 1.038*** |  | < 0.001 |  | 1.090 |  | < 0.001 |  |
| LR-test chi-square | 5.464 |  | 0.141 |  | 7.148 |  | 0.067 |  |
| Pseudo R-squared | 0.003 |  |  |  | 0.003 |  |  |  |
| **Same screening participation** | **Estimate** | ***SE*** | ***p*** | ***OR*** | **Estimate** | ***SE*** | ***p*** | ***OR*** |
| Tie distance 2 | -0.073 | 0.185 | 0.693 | 0.930 | -0.591*** | 0.169 | < 0.001 | 0.554 |
| Tie distance 3 | 0.160 | 0.238 | 0.500 | 1.174 | -0.574*** | 0.162 | < 0.001 | 0.563 |
| Tie distance 4 | 0.186 | 0.258 | 0.470 | 1.205 | -0.480** | 0.154 | 0.002 | 0.619 |
| SD random intercept | 0.886*** |  | < 0.001 |  | < 0.001 |  | 1.000 |  |
| LR-test chi-square | 1.844 |  | 0.605 |  | 10.732* |  | 0.013 |  |
| Pseudo R-squared | 0.001 |  |  |  | 0.004 |  |  |  |
| **Same self-sample^1^** | **Estimate** | ***SE*** | ***p*** | ***OR*** | **Estimate** | ***SE*** | ***p*** | ***OR*** |
| Tie distance 2 | -0.313 | 0.360 | 0.385 | 0.731 | -0.165 | 0.321 | 0.607 | 0.848 |
| Tie distance 3 | 0.344 | 0.549 | 0.531 | 1.411 | -0.586 | 0.303 | 0.053 | 0.556 |
| Tie distance 4 | -0.109 | 0.638 | 0.864 | 0.896 | -0.748* | 0.299 | 0.012 | 0.473 |
| SD random intercept | 0.809 |  | 0.916 |  | < 0.001 |  | 1.000 |  |
| LR-test chi-square | 2.192 |  | 0.534 |  | 9.430* |  | 0.024 |  |
| Pseudo R-squared | 0.005 |  |  |  | 0.011 |  |  |  |
| **Same knowledge level** | **Estimate** | ***SE*** | ***p*** | ***OR*** | **Estimate** | ***SE*** | ***p*** | ***OR*** |
| Tie distance 2 | -0.208 | 0.175 | 0.234 | 0.812 | -0.250 | 0.166 | 0.132 | 0.779 |
| Tie distance 3 | -0.045 | 0.228 | 0.842 | 0.956 | -0.239 | 0.160 | 0.136 | 0.788 |
| Tie distance 4 | -0.178 | 0.240 | 0.458 | 0.837 | -0.184 | 0.153 | 0.230 | 0.832 |
| SD random intercept | 0.449* |  | 0.021 |  | < 0.001 |  | 1.000 |  |
| LR-test chi-square | 1.875 |  | 0.599 |  | 2.694 |  | 0.441 |  |
| Pseudo R-squared | 0.001 |  |  |  | 0.001 |  |  |  |
| **Same attitude** | **Estimate** | ***SE*** | ***p*** | ***OR*** | **Estimate** | ***SE*** | ***p*** | ***OR*** |
| Tie distance 2 | -0.016 | 0.188 | 0.932 | 0.984 | 0.348 | 0.216 | 0.107 | 1.416 |
| Tie distance 3 | 0.006 | 0.241 | 0.982 | 1.006 | 0.424 | 0.222 | 0.056 | 1.528 |
| Tie distance 4 | 0.184 | 0.265 | 0.487 | 1.202 | 0.034 | 0.220 | 0.877 | 1.034 |
| SD random intercept | 1.048*** |  | < 0.001 |  | 1.260*** |  | < 0.001 |  |
| LR-test chi-square | 0.640 |  | 0.887 |  | 14.373** |  | 0.002 |  |
| Pseudo R-squared | < 0.001 |  |  |  | 0.006 |  |  |  |
| **Same intention** | **Estimate** | ***SE*** | ***p*** | ***OR*** | **Estimate** | ***SE*** | ***p*** | ***OR*** |
| Tie distance 2 | -0.138 | 0.203 | 0.497 | 0.871 | -0.401 | 0.224 | 0.074 | 0.670 |
| Tie distance 3 | -0.265 | 0.253 | 0.296 | 0.768 | -0.202 | 0.229 | 0.377 | 0.817 |
| Tie distance 4 | -0.290 | 0.275 | 0.292 | 0.748 | -0.048 | 0.228 | 0.832 | 0.953 |
| SD random intercept | 1.627*** |  | < 0.001 |  | 1.324*** |  | < 0.001 |  |
| LR-test chi-square | 1.551 |  | 0.671 |  | 8.986* |  | 0.029 |  |
| Pseudo R-squared | 0.001 |  |  |  | 0.003 |  |  |  |
| **Same IDM** | **Estimate** | ***SE*** | ***p*** | ***OR*** | **Estimate** | ***SE*** | ***p*** | ***OR*** |
| Tie distance 2 | -0.256 | 0.189 | 0.176 | 0.774 | -0.197 | 0.200 | 0.324 | 0.821 |
| Tie distance 3 | 0.275 | 0.252 | 0.274 | 1.317 | -0.156 | 0.205 | 0.448 | 0.856 |
| Tie distance 4 | -0.213 | 0.269 | 0.430 | 0.808 | -0.102 | 0.204 | 0.618 | 0.903 |
| SD random intercept | 0.870*** |  | < 0.001 |  | 0.779** |  | 0.003 |  |
| LR-test chi-square | 7.272 |  | 0.064 |  | 1.335 |  | 0.721 |  |
| Pseudo R-squared | 0.004 |  |  |  | < 0.001 |  |  |  |
| *Note*. Reference category of predictor ‘tie distance’ = tie distance 1.  Estimate = logit *B*-coefficient for the model predictors and between-group *SD* for the random intercept, *SE* = standard error, *p* = *p*-value, *OR* = odds ratio, IDM = informed decision-making.  * *p* < 0.05, ** *p* < 0.01, *** *p* < 0.001  ^1^ sample size Moroccan-Dutch sample = 352 pairs within 67 trees; sample size Turkish-Dutch sample = 645 pairs within 43 trees. | | | | | | | | |
